# Dehydration is associated with production of organic osmolytes and predicts physical long-term symptoms after COVID-19: a multicenter cohort study

**DOI:** 10.1186/s13054-022-04203-w

**Published:** 2022-10-21

**Authors:** Michael Hultström, Miklos Lipcsey, Dave R. Morrison, Tomoko Nakanishi, Guillaume Butler-Laporte, Yiheng Chen, Satoshi Yoshiji, Vincenzo Forgetta, Yossi Farjoun, Ewa Wallin, Ing-Marie Larsson, Anders Larsson, Adriana Marton, Jens Marc Titze, Sandra Nihlén, J. Brent Richards, Robert Frithiof

**Affiliations:** 1 Anaesthesiology and Intensive Care Medicine, Department of Surgical Sciences, Uppsala University, Akademiska Sjukhuset, ANOPIVA, Ing70, 2Tr, 75185 Uppsala, Sweden; 2 Integrative Physiology, Department of Medical Cell Biology, Uppsala University, Uppsala, Sweden; 3 Department of Epidemiology, Biostatistics and Occupational Health, McGill University, Montreal, QC Canada; 4 Lady Davis Institute of Medical Research, Jewish General Hospital, McGill University, Montreal, QC Canada; 5 Hedenstierna Laboratory, Department of Surgical Sciences, Uppsala University, Uppsala, Sweden; 6 Department of Human Genetics, McGill University, Montreal, QC Canada; 7 Kyoto-McGill International Collaborative Program in Genomic Medicine, Graduate School of Medicine, Kyoto University, Kyoto, Japan; 8 Japan Society for the Promotion of Science, Tokyo, Japan; 9 Clinical Chemistry, Department of Medical Sciences, Uppsala University, Uppsala, Sweden; 10 Program in Cardiovascular and Metabolic Disorders, Duke- NUS Medical School, Singapore, Singapore; 11 Division of Nephrology, Duke University Medical Center, Durham, NC USA; 12 Department of Twin Research, King’s College London, London, UK; 13 5 Prime Sciences, Montreal, QC Canada; 14 The Broad Institute of Harvard and MIT, Cambridge, MA USA; 15 Fulcrum Genomics, Bolder, CO USA

**Keywords:** Aestivation, Acute kidney injury, Intensive care medicine, SARS-CoV-2, Urea synthesis, Long-COVID

## Abstract

**Background:**

We have previously shown that iatrogenic dehydration is associated with a shift to organic osmolyte production in the general ICU population. The aim of the present investigation was to determine the validity of the physiological response to dehydration known as aestivation and its relevance for long-term disease outcome in COVID-19.

**Methods:**

The study includes 374 COVID-19 patients from the Pronmed cohort admitted to the ICU at Uppsala University Hospital. Dehydration data was available for 165 of these patients and used for the primary analysis. Validation was performed in Biobanque Québécoise de la COVID-19 (BQC19) using 1052 patients with dehydration data. Dehydration was assessed through estimated osmolality (eOSM = 2Na + 2 K + glucose + urea), and correlated to important endpoints including death, invasive mechanical ventilation, acute kidney injury, and long COVID-19 symptom score grouped by physical or mental.

**Results:**

Increasing eOSM was correlated with increasing role of organic osmolytes for eOSM, while the proportion of sodium and potassium of eOSM were inversely correlated to eOSM. Acute outcomes were associated with pronounced dehydration, and physical long-COVID was more strongly associated with dehydration than mental long-COVID after adjustment for age, sex, and disease severity. Metabolomic analysis showed enrichment of amino acids among metabolites that showed an aestivating pattern.

**Conclusions:**

Dehydration during acute COVID-19 infection causes an aestivation response that is associated with protein degradation and physical long-COVID.

*Trial registration*: The study was registered à priori (clinicaltrials.gov: NCT04316884 registered on 2020-03-13 and NCT04474249 registered on 2020-06-29).

**Graphical abstract:**

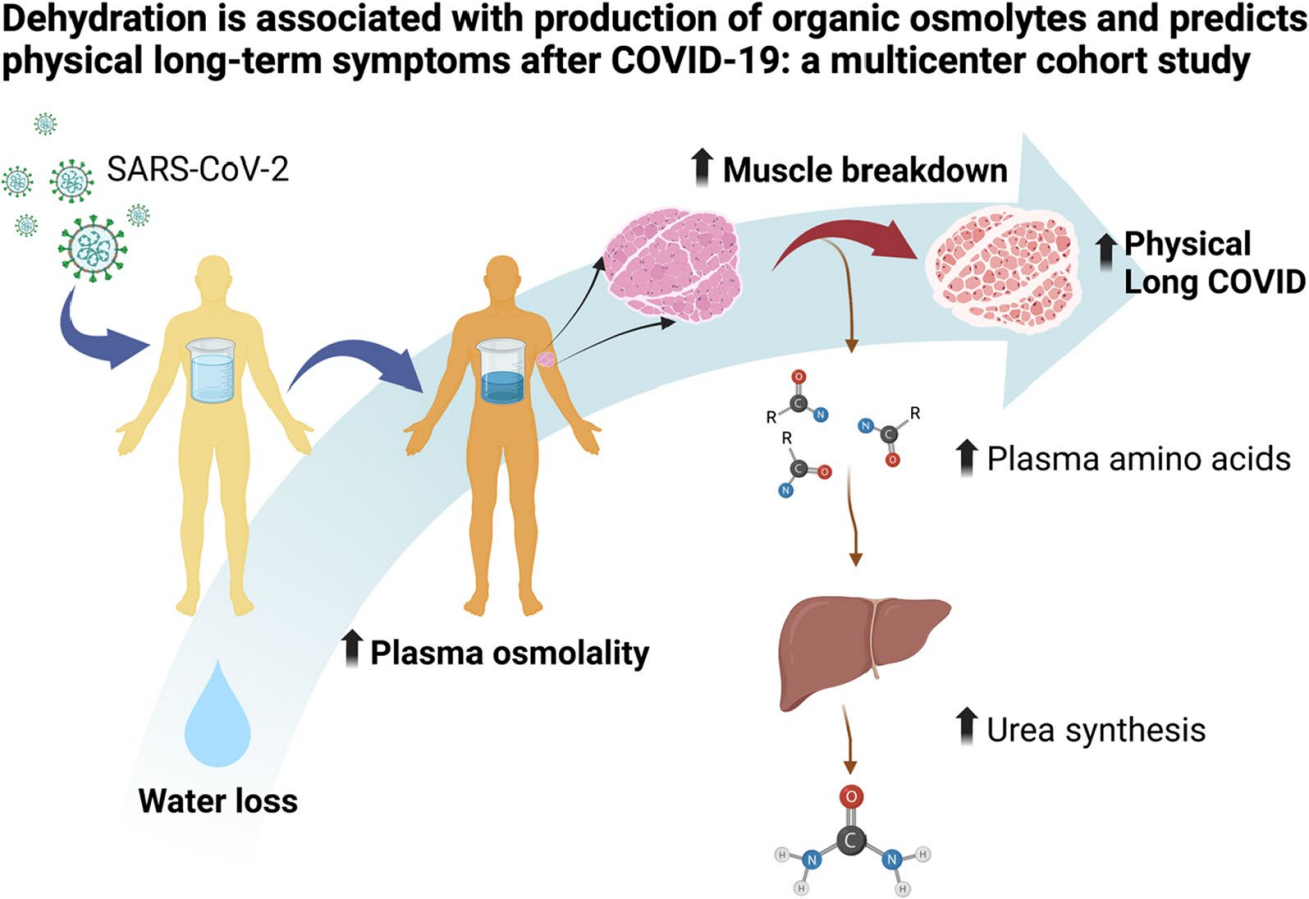

**Supplementary Information:**

The online version contains supplementary material available at 10.1186/s13054-022-04203-w.

## Introduction

Severe coronavirus infectious disease 2019 (COVID-19) has been associated with dehydration[1], which has been directly tied to oliguric acute kidney injury (AKI) [2], and early dehydration has been proposed as a mechanism of more severe disease [3]. We have previously shown that iatrogenic dehydration during treatment in the intensive care unit (ICU) is associated with a shift to organic osmolyte production in the general ICU population [4]. This is part of a response to dehydration that is conserved throughout evolution from aestivating worms to higher animals and even mammals [5], and is consistent with the literature on cellular metabolism during dehydration showing protein breakdown in a variety of cell types induced by cell shrinkage [6]. The components of aestivation in mammals include osmolyte synthesis from amino acids, urine concentration, and skin hypoperfusion. This, in turn, causes increased plasma urea and plasma glucose, oliguria, peripheral coldness, catabolic muscle mass loss, and increased total peripheral resistance with a concomitant increase in blood pressure [7–9].

We found the aestivation concept highly consistent with the clinical presentation of critically ill COVID-19 patients who show a remarkable circulatory stability despite an inflammatory response reminiscent of sepsis and present with primarily oliguric acute kidney injury [2] with disproportionately high urea compared to creatinine. Finally, many critically ill COVID-19 patients required insulin treatment to control plasma glucose despite not having diabetes mellitus beforehand [10]. Fluid removal with concomitant dehydration is a common treatment strategy in acute respiratory distress syndrome (ARDS). During the SARS-CoV-2 pandemic restrictive fluid management and active deresuscitation have been part of clinical guidelines since early in the pandemic [11]. Taken together this made us hypothesize that dehydration, in the form of relative water loss, due to fever and reduced intake during the early illness, and iatrogenic restrictive fluid therapy during the critical illness, cause an aestivation response in critically ill COVID-19 patients.

**Fig. 1:**
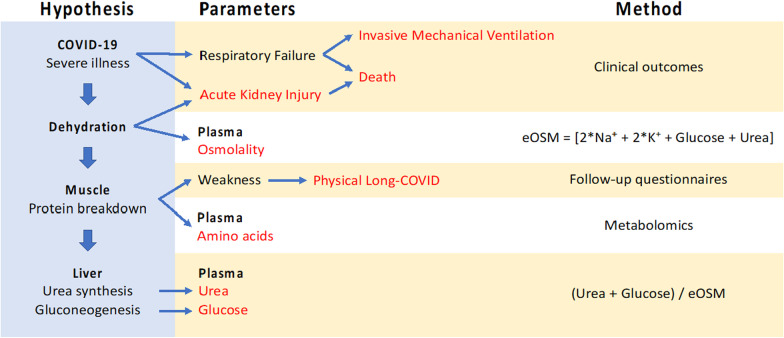
The aestivation hypothesis, which is the subject of the present investigation, is that there is a metabolic response to dehydration that induces the production of the organic osmolytes urea and glucose. This requires protein breakdown, which is further hypothesized to cause physical long-COVID because of the muscle weakness. The main parameters investigated in the present study are estimated osmolality (eOSM = 2*Na^+^ + 2*K^+^ + Glucose + Urea) that is used as a measure of dehydration. The degree of aestivation is measure as the decreasing proportion of ionic (sodium and potassium) osmolytes to total osmolality, and increasing proportion of organic osmolytes (glucose and urea). Finally, the metabolic shift to protein breakdown is investigated using plasma metabolomics to detect release of amino acids that will, in turn, be used by the liver for gluconeogenesis and urea synthesis

The aim of this study was to investigate the presence of an aestivation reaction in response to dehydration in COVID-19 patients from two independent cohorts, and to determine whether this was associated with organ failure during intensive care, death, or specific clusters of post-acute COVID symptoms. Our hypothesis was that dehydration would correlate with organic osmolyte production and protein breakdown, which would be associated with physical long-COVID because of muscle weakness (Fig. 1), but not mental long-COVID in the form of anxiety, depression, or pain.

## Methods

**Fig. 2:**
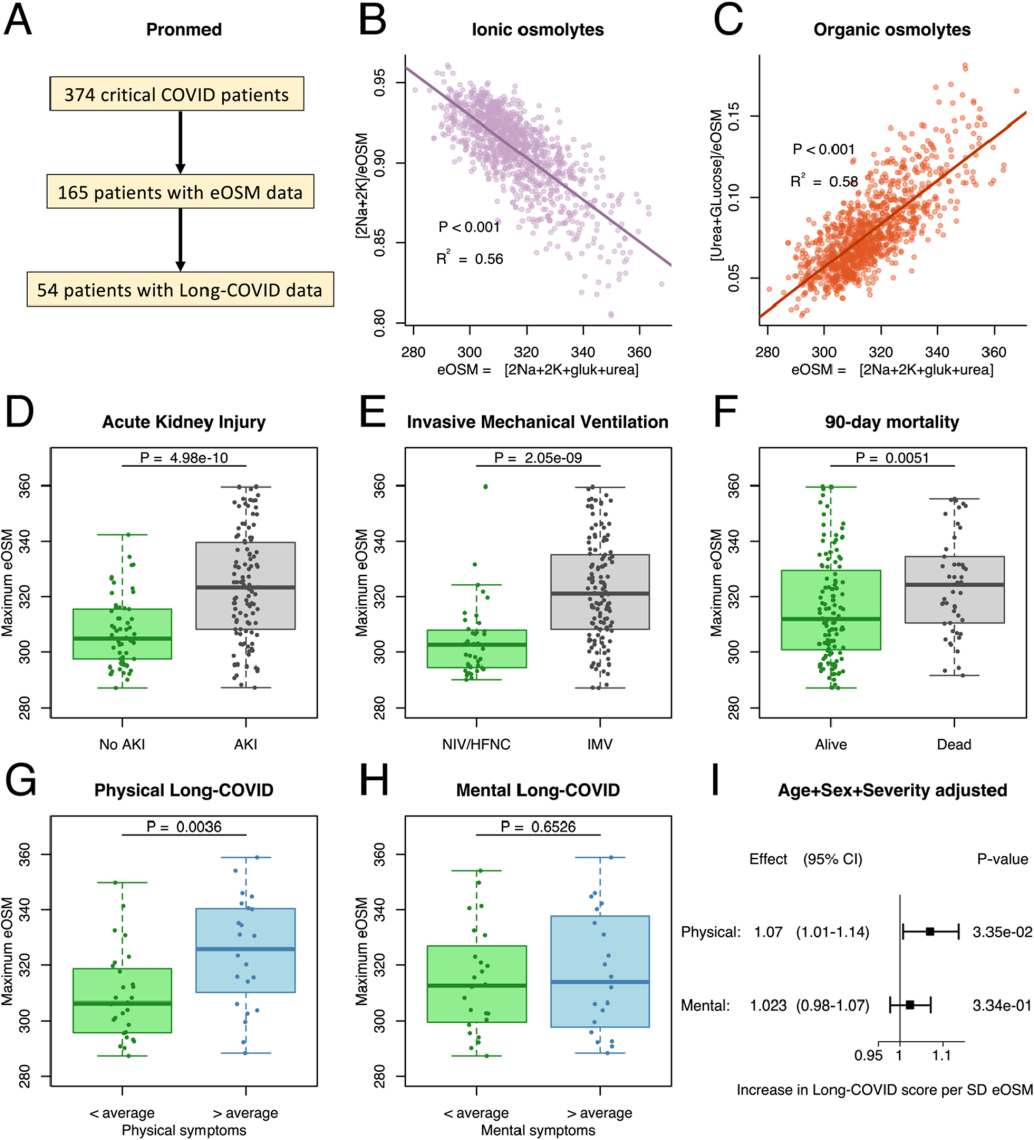
**A** The present study was based on a discovery analysis of the prospective observational cohort Pronmed with critically ill COVID-19 patients from the ICU at Uppsala University Hospital (**A**). Out of a total of 374 included patients, 165 had plasma urea analyzed at some point during their ICU stay and could be used to analyze the aestivation response through estimated osmolality (eOSM). Out of these 54 participated in the 3–6 month follow-up and provided data on remaining symptoms. **B**, **C** Aestivation response showing the fraction of the estimated total osmolality (eOSM = 2*Na + 2*K + Glucose + Urea) for the ionic osmolytes sodium and potassium (**B**), and the organic osmolytes glucose and urea (**C**), showing a shift to organic osmolytes with increasing eOSM. Pearson regression line with *p* value and R^2^ for the correlation. **D**, **E**, **F** Maximum dehydration measured as eOSM was higher in critically ill COVID-19 patients with important outcomes such as acute kidney injury (AKI, **D**), need for invasive mechanical ventilation (IMV, **E**), and 90-day mortality (**F**). Student’s T-test was used to test for chance difference. **G**, **H**, **I** Interestingly, maximal dehydration was higher in patients who reported more than average remaining physical (**G**) symptoms after 3–6 months, but not mental symptoms (**H**). Student’s T-test was used to test for chance difference. The analysis adjusted for sex, age and disease severity using SAPS-3 shows a substantial effect of eOSM on physical long-COVID but not on mental long-COVID (**I**)

The discovery set was taken from the Pronmed cohort that consists of 374 critically ill COVID-19 patients admitted to the intensive care unit (ICU) at Uppsala University Hospital, in Uppsala, Sweden between March 13, 2020 and June 30, 2021 (Fig. 2A). Patients were offered to participate in a follow-up study three to six months after discharge from ICU with questionnaires and a personal visit.

**Fig. 3:**
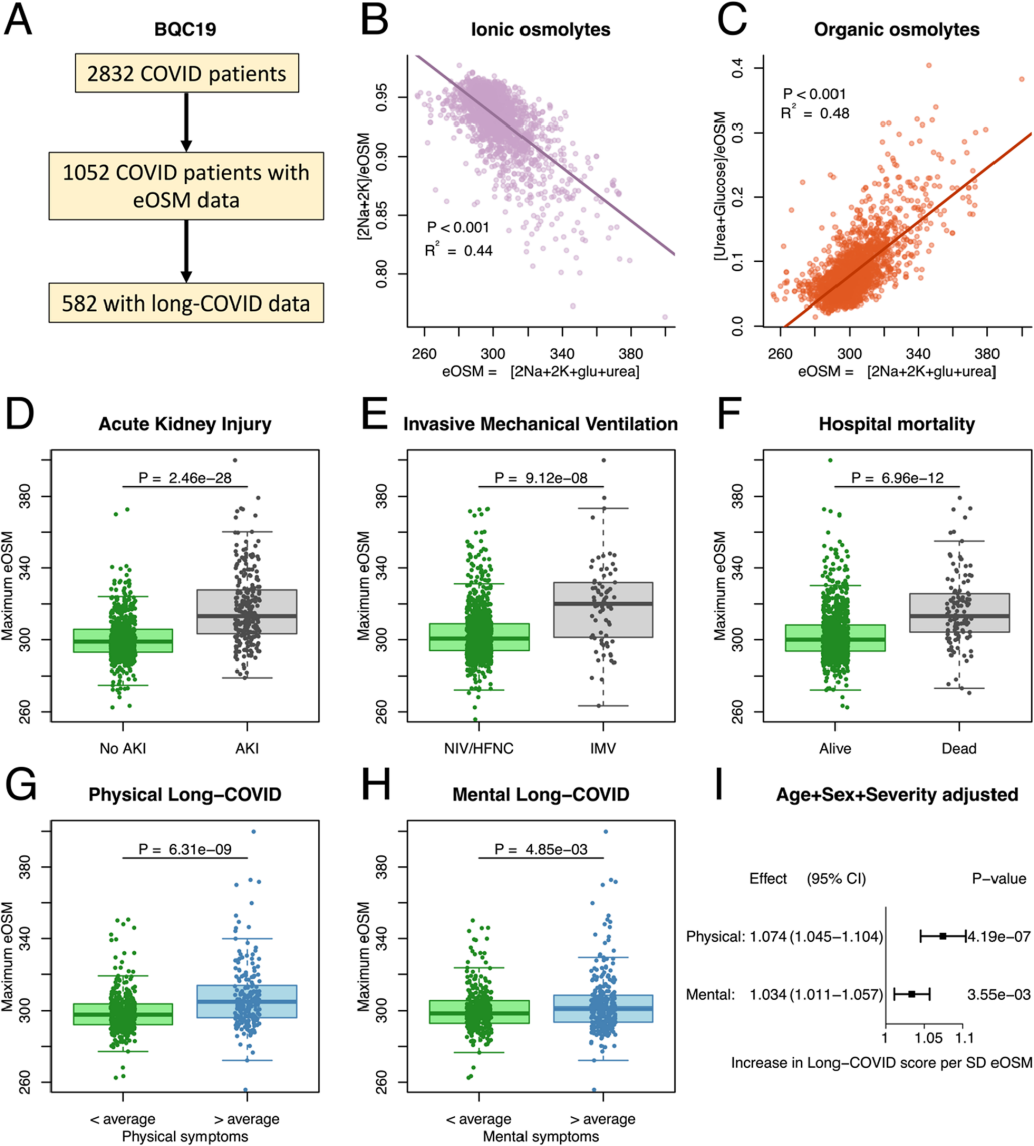
**A** To validate the findings the same analysis was performed in the Biobanque Québécoise de la COVID-19 (BQC19) (**A**). Out of a total of 3768 included patients, 1637 had plasma urea analyzed at some point during their ICU stay and could be used to analyze the aestivation response through estimated osmolality (eOSM). Out of these 872 patients filled out questionnaires on remaining symptoms. **B**, **C** The validation cohort recapitulates the aestivation response for the ionic osmolytes (**B**), and the organic osmolytes (**C**). Pearson regression line with *p* value and R^2^ for the correlation. D,E,F: As in the Pronmed cohort maximum dehydration was higher in more severely ill COVID-19 patients for the outcomes acute kidney injury (AKI, **D**), need for invasive mechanical ventilation (IMV, **E**), and hospital mortality (**F**). Student’s T-test was used to test for chance difference. **G**, **H**, **I** Finally, the association of maximal dehydration with physical symptoms after 3–6 months was reproduced (**G**). In addition, an association was seen for mental long-COVID in the BQC19 cohort (**H**). Student’s T-test was used to test for chance difference. The analysis adjusted for sex, age and disease severity using ICU-admission reproduces the substantially greater effect eOSM has on physical long-COVID than on mental long-COVID, although both show significant effect (**I**)

The validation set was taken from the Biobanque Québécoise de la COVID-19 (BQC19) release 5 cohort that consist of 3768 patients from 10 hospitals in Quebéc, Canada between January 12, 2020 and December 12, 2021 (Fig. 3A). It is a hospital-based prospective cohort enrolling participants with PCR proven SARS-CoV-2 infection (*N* = 2832), and controls who presented to hospital with signs or symptoms consistent with COVID-19, but with negative PCR tests for SARS-CoV-2 (*N* = 824). Patients with indeterminate tests were excluded (*N* = 112). Patients were invited for post-acute follow-up at 1, 3, 6, 12, and 18 months after the enrolment.

The degree of dehydration was measured as increasing estimated plasma osmolality calculated as:

$${\text{eOSM}} = {\text{2Na}}^{ + } + {\text{2K}}^{ + } + {\text{Glucose}} + {\text{Urea}}$$

The proportion total osmolality caused by ionic osmolytes as the ratio of sodium plus potassium to eOSM: (2Na^+^ + 2K^+^)/(2Na^+^ + 2K^+^ + Glucose + Urea). The proportion due to organic osmolytes as the ratio of glucose plus urea to eOSM (Glucose + Urea/(2Na^+^ + 2K^+^ + Glucose + Urea).

Long COVID symptoms were grouped into the major clusters physical or mental based on the instruments used in the two cohorts. A continuous long-COVID score between 0 and 1 was calculated by scaling each instrument to a 0 to 1 score and calculating the average of all scores in each cluster (Additional file 1: Table S1). The instruments used in Pronmed were the Montreal Cognitive Assessment (MOCA), Euro-Quality-of-Life Group 5-dimension, 5-level (EQ-5D-5L), Patient Health Questionnaire 9-question (PHQ-9, a depression instrument), and the Generalized Anxiety Disorder 7-question (GAD-7) questionnaires. In BQC19 a custom post-COVID questionnaire was used. Instruments with decreasing score for increasing symptoms such as MOCA and PHQ9 used in Pronmed were reversed by subtracting the score from the maximal score before scaling. The effect of eOSM on physical and mental long-COVID was estimated using the continuous score. To illustrate the difference in eOSM in patients with more or less severe long-COVID for either physical or mental symptoms the mean of the continuous score was used as cut-off.

Plasma metabolomic analysis was available for 575 patients in the BQC19 cohort with a total of 1435 detected metabolites. Metabolites that showed an aestivation-like response expressed as a positive correlation of the metabolite / eOSM to eOSM were defined as aestivating metabolites. This indicates that the aestivating metabolites increase in concentration more than would be expected from the increase in osmolality alone. The metabolic pathways involved in the aestivation response was identified through enrichment analysis using KEGG-pathways excluding poorly annotated metabolites and xenometabolites leaving 797 metabolites in the analysis.

### Statistics

Statistics were calculated using R version 4.0.5. Data are reported as mean (SD) or frequency (%). Pearson’s correlation was used for correlating the proportion of ionic and organic osmolytes as well as metabolites to total osmolality. Student’s T-test was used to test difference of eOSM between patients with and without acute outcomes, and with long-COVID score above or below the average score. Multivariable linear regression was used to analyze the effect of increased eOSM on physical and mental long-COVID with correction for age, sex and severity. In Pronmed, severity was adjusted for using the simplified acute physiology score 3 (SAPS3) [12] commonly used in intensive care, while in the general population of BQC19 was adjusted for using ICU admission. The area under the receiver operating characteristic (ROC) curve was used to evaluate the importance for eOSM in predicting long-term outcomes using an 80:20 split of the BQC dataset correcting for age, sex and severity. A sensitivity analysis using only the clinical variables age, sex and severity was performed and the Bayesian information criterion (BIC) was used to distinguish the better model. Metabolomic correlation was calculated to identify metabolites that showed an aestivation pattern where the ratio of the metabolite to total eOSM increased with increasing eOSM. The analysis was adjusted using Bonferroni’s correction for the number of metabolites, an adjusted *p* value < 0.05 was considered significant. Enrichment of metabolic pathways with aestivating behaviour was calculated using the exact binomial test. *p* < 0.05 was accepted as statistically significant.

## Results

The Pronmed cohort consisted of 374 patients out of which 165 had data on all of the osmolytes, 54 of these patients completed follow-up after 3–6 months (Fig. 2A). The average age was 63 ± 14 years and 25.5% were women (Table 1). The BQC19 cohort consisted of 2832 COVID-19 patients, of whom 1052 had osmolyte data, and 582 completed follow-up questionnaires (Fig. 3A). The average age was 63 ± 20 years and 54.4% were women (Table 1). The main differences between the cohorts is that Pronmed was recruited only from ICU admissions, while BQC19 was recruited from emergency room visits, and that Pronmed follow-up was 3–6 months after discharge from the ICU while BQC19 follow-up was considerably earlier. Therefore, BQC includes less severely ill, non-hospitalized patients, and have a lower rate of acute endpoints (Table 1). This also shows in the closer of sex in the BQC19-cohort, since women are at lower risk of severe disease. On the other hand, the earlier follow-up may still lead to similar levels of long-COVID despite lower acute severity.

**Table 1:** Clinical characterization of the Pronmed cohort analyzed as the discovery set, and the Biobanque Québécoise de la COVID-19 (BQC19) cohort used for validation

	Pronmed		BQC19	
n	165	Missing (%)	1052	Missing (%)
Women (%)	42 (25.5)	0	572 (54.4)	0
Age (mean ± SD)	63 ± 14	0	63 ± 20	2 (0.2)
max eOSM (mean ± SD)	318 ± 19	0	304 ± 16	0
Hospitalized (%)	165 (100)	0	885 (90.6)	75 (7.1)
ICU admission (%)	165 (100)	0	304 (34.7)	9 (0.9)
Invasive ventilation (%)	126 (76.4)	0	74 (7.2)	21 (2.0)
Acute kidney injury (%)	106 (64.6)	1 (0.6)	238 (24.5)	8 (0.8)
Death (%)	49 (29.9)	1 (0.6)	122 (12.6)	81 (7.7)

Long-COVID				
n	54	Missing (%)	615	Missing (%)
Time to Follow-up (median[IQR])	139 [123–181]	0	101 [66–184]	0
Physical (mean ± SD)	0.28 ± 0.20	3 (5.5)	0.24 ± 0.30	33 (5.4)
Mental (mean ± SD)	0.23 ± 0.16	10 (18)	0.20 ± 0.22	16 (2.6)

Total number of patients correspond to those with lab data on dehydration, total number of long COVID corresponds to the number of patients with lab data that also completed any follow-up. Long COVID is expressed as a mean score between zero and one for questionnaire parameters pertaining to either physical or mental remaining symptoms. SD: Standard Deviation, IQR: inter quartile range

The aestivation results in the two cohorts are entirely consistent and show that increasing eOSM is correlated with decreasing importance of the ionic osmolytes sodium and potassium (Figs. 1B and 3B), while an increasing role of the organic osmolytes urea and glucose (Fig. 1C and 3C). This is consistent with the aestivation response where dehydration with increased osmolality drives production of organic osmolytes through gluconeogenesis and urea synthesis.

**Fig. 4:**
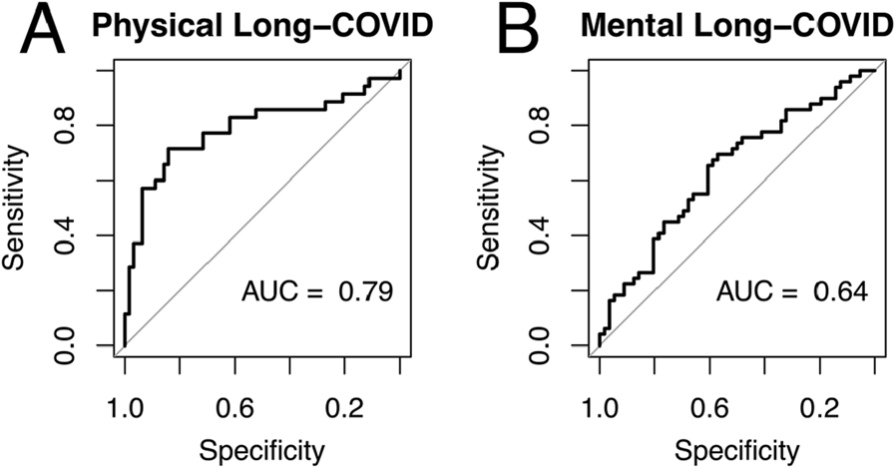
Predictive validation was performed using an 80:20 split of the BQC19 dataset to test the accuracy of the effect of dehydration adjusted for age, sex and disease severity using ICU-admission. Maximal dehydration measured as estimated osmolality (eOSM = 2*Na^+^ + 2*K^+^ + Glucose + Urea) was a stronger predictor of physical long-COVID (**A**) as indicated by larger area under the ROC curve (AUC) than for mental long-COVID (**B**)

Dehydration in the form of increased eOSM was strongly associated with disease severity indicated by association with acute outcomes in the form of acute kidney injury (AKI, Figs. 2D and 3D), invasive mechanical ventilation (IMV, Figs. 2E and 3E) and death (Figs. 2F and 3F). However, for long-term outcomes dehydration was primarily associated with physical symptoms of long-COVID (Figs. 2G and 3G) indicating possible consequences of protein degradation in the form of muscle wasting. Mental long-COVID defined by symptoms of depression, anxiety or pain was not associated with dehydration in Pronmed (Fig. 2H), but showed a significant effect in BQC19 (Fig. 3H). Using multivariable linear regression to correct for age, sex and disease severity while taking advantage of the continuous score for long-COVID verified a substantially larger effect of maximal eOSM on physical long-COVID than on mental long-COVID with comparable effect sizes between the cohorts (Figs. 2I and 3I). Further, the area under the curve (AUC) of the receiver operating characteristic (ROC) curve (Figs. 4A, B) indicates excellent performance of maximal eOSM for predicting physical long-COVID, but weaker performance for mental long-COVID. In a sensitivity analysis the model including maximal eOSM showed a lower BIC compared to a model with only clinical variables (95 compared to 113), indicating a better performance even with the collinearity between dehydration and severity.

Finally, analysis of the plasma metabolome in 575 patients from the BQC19 cohort identified 274 metabolites that showed an aestivation phenotype with an increasing ratio of the metabolite to total eOSM with increasing eOSM. Pathway analysis identified amino acid metabolism as strongly enriched, which is consistent with protein breakdown in muscle and release of amino acids for urea synthesis in the liver (Table 2).

**Table 2:** Plasma metabolomic analysis of 575 patients from the Biobanque Québécoise de la COVID-19 (BQC19) identified 1435 different metabolites

Super Pathway	Total	Aestivation correlated	Expected	Enrichment (%)	Enriche ment *p* value
Amino Acid	211	123	73	68	3.37e−36
Peptide	32	20	11	82	8.49e−08
Nucleotide	34	20	12	67	3.52e−07
Carbohydrate	25	13	9	44	0.00023
Cofactors and Vitamins	39	13	13	0	0.38
Energy	10	1	3	− 67	0.70
Lipid	446	84	153	− 45	0.95

Pathway enrichment analysis of the plasma metabolome identified 274 metabolites that correlated with aestivation after Bonferroni correction. KEGG Pathway enrichment analysis identified amino acid metabolites as significantly enriched in plasma indicating protein degradation as the major source of nitrogen used for urea synthesis during dehydration

## Discussion

The main finding of the present study is that COVID-19 patients show metabolic aestivation in response to dehydration, which is associated with disease severity. The main component of aestivation is urea synthesis and gluconeogenesis, probably with muscle catabolism as the main source of protein as indicated by increased plasma amino acid metabolites. This may be a pathogenic mechanism of weakness and fatigue as part of the post-acute syndrome widely reported in COVID-19 patients.

This study extends the previously published literature on several fronts. It presents the largest clinical cohort where aestivation has been studied and is the first to show strong associations with long-term physical symptoms. It is the only clinical study of dehydration in COVID-19. In addition, it is the first to show dehydration associated protein catabolism using plasma metabolomics with results consistent to previously published experimental data [7, 9]. The results are consistent with our previously published cohort of patients without COVID-19 [4], which indicates generalizability of the aestivation concept during critical illness. However, the results pertaining to long-term symptoms and plasma metabolomics have only been performed in the present study and need confirmation in the non-COVID setting.

The dehydration in itself is probably caused by two main mechanisms. Firstly, dehydration because of several days of fever and reduced water intake during the initial illness, and secondly, iatrogenic dehydration as part of clinical management. This fits well with the strong association between severity and mortality and eOSM. Because of the strong association between severity and eOSM the present data is not sufficient to disentangle to what degree disease severity causes dehydration that, in turn, causes urea synthesis with protein breakdown, and to what degree disease severity causes protein breakdown by other means. Further, data resolution over time is not sufficient to determine whether dehydration develops before or after the acute outcomes such as AKI or ICU admission. While earlier data from diverse models of dehydration indicate that protein breakdown is caused by dehydration independently of COVID-19 [4, 7–9] or other acute disease severity [4], the question of causality in COVID-19 and in ICU in general will require new randomized trials to compare dehydration to normoosmolality in patients with similar disease severity. In the present analysis we can only note that eOSM provides some additional information beyond clinical variables only as indicated by a lower BIC in sensitivity analysis.

The strengths of the present study are that it is the first to analyze the presence of an aestivation response in COVID-19 patients and the first to provide a long-term follow-up of both mortality and long-term symptoms associated with dehydration. A weakness is the observational nature of the study that relies on clinically indicated analysis of urea, which was only performed in part of the cohorts introducing a potential selection bias, in particular for the Pronmed cohort. However, in BQC19 urea was analysed in the emergency room in almost all patients making the risk of bias in the validation cohort much smaller. A further limitation is the lack of detailed information on nutritional intake, which could bias the balance between ionic and organic osmolytes. However, we have previously found that this associates only weakly with the aestivation phenotype [4]. In addition, the report forms, questionnaires, and follow-up times used in the two cohorts were not the same, making the definitions of acute and long-term symptoms slightly different between the cohorts. However, the major outcomes and symptom clusters used for long-COVID were harmonized and show similar results and effect sizes indicating robustness of the aestivation phenotype despite minor variations in the definition of endpoints.

In conclusion, humans show a robust shift to organic osmolyte production in response to dehydration during severe illness. In COVID-19, this aestivation response is strongly associated with more severe acute disease as well as remaining physical symptoms after recovery. The main implication for future trials is that any investigation of long-term physical outcome of severe illness may benefit from controlling for dehydration in their analysis. Further, new randomized controlled trials of targeting normal plasma osmolality during deresuscitation should be performed. This may be achieved by allowing administration of free water in conjunction with diuretics to allow for sodium mobilization without hyperosmolality during deresuscitation.

## Supplementary Information

**Additional file 1**. Clinical endpoints in Pronmed and Biobanque Québécoise de la COVID-19 (BQC19) for outcomes of acute COVID-19 and Long COVID-19 clustered as physical or mental symptoms.

## Data Availability

Discovery cohort data is available through the SciLifeLab data repository after securing ethical permission and appropriate data access agreements (https://doi.org/10.17044/scilifelab.14229410). Validation cohort data is available through the Biobanque Québécoise de la COVID-19 (BQC19) after securing ethical permission and appropriate data access agreements (https://www.quebeccovidbiobank.ca).
