# Supplementary material for: Dehydration is associated with production of organic osmolytes and predicts physical long-term symptoms after COVID-19: a multicenter cohort study
Source: Crit Care. 2022 Oct 21;26:322. doi: 10.1186/s13054-022-04203-w (PMC9585783; doi:10.1186/s13054-022-04203-w)
Supplement: Supplementary file 1 — Additional file 1. Clinical endpoints in Pronmed and Biobanque Québécoise de la COVID-19 (BQC19) for outcomes of acute COVID-19 and Long COVID-19 clustered as physical or mental symptoms. [file 13054_2022_4203_MOESM1_ESM.docx]

**Supplement to: Dehydration is associated with production of organic osmolytes and predicts physical long-term symptoms after COVID-19: a multicenter cohort study**

**Table S1**

| **Pronmed** |  |  |  |
| --- | --- | --- | --- |
| **Acute COVID outcomes** | **Criteria** |  | **Scale** |
| Acute kidney injury | Creatinine based according to KDIGO | | Yes/No |
| Invasive mechanical ventilation | Clinical diagnosis in CRF | | Yes/No |
| Death | 90-day mortality | | Yes/No |
| **Physical Long-COVID** | **Instrument** | **Score** | **Normalised scale** |
| Mobility | EQ5D5L | 1 to 5 | 0 to 1 |
| Self-Care | EQ5D5L | 1 to 5 |  |
| Usual Activities | EQ5D5L | 1 to 5 |  |
| Physical Fatigue | MFI | 4 to 20 |  |
| **Mental Long-COVID** | **Instrument** | **Score** | **Normalised scale** |
| Pain | EQ5D5L | 1 to 5 | 0 to 1 |
| Discomfort | EQ5D5L | 1 to 5 |  |
| Mental Fatigue | MFI | 4 to 20 |  |
| Depression | PHQ9 | 0 to 27 |  |
| Anxiety | GAD7 | 0 to 21 |  |
| Cognitive dysfunction | MOCA | 30 to 0 |  |
| **BQC19** |  |  |  |
| **Acute COVID outcomes** |  |  | **Scale** |
| Acute kidney injury | Clinical diagnosis in CRF | | Yes/No |
| Invasive mechanical ventilation | Clinical diagnosis in CRF | | Yes/No |
| Death | In-hospital mortality in CRF | | Yes/No |
| **Physical Long-COVID** | **Instrument** | **Score** | **Normalised scale** |
| Mobility | BQC19 | 1 to 5 | 0 to 1 |
| Self-Care | BQC19 | 1 to 5 |  |
| Usual Activities | BQC19 | 1 to 5 |  |
| Carrying | BQC19 | 1 to 3 |  |
| Walking | BQC19 | 1 to 3 |  |
| Climbing Stairs | BQC19 | 1 to 3 |  |
| Rising out of a Chair | BQC19 | 1 to 3 |  |
| **Mental Long-COVID** | **Instrument** | **Score** | **Normalised scale** |
| Pain and Discomfort | BQC19 | 1 to 5 | 0 to 1 |
| Anxiety and Depression | BQC19 | 1 to 5 |  |

Clinical endpoints used for primary analysis in Pronmed and validation using Biobanque Québécoise de la COVID-19 (BQC19) for outcomes of acute COVID-19 and Long COVID-19 clustered as physical or mental symptoms using a continuous score that was normalized to zero to one and averaged for physical and mental symptoms for each of the cohorts to harmonize the analysis. KDIGO: Kidney Disease Improving Global Outcomes, CRF: Clinical Report Form, EQ5D5L: Five-dimensional Quality of life questionnaire. MFI: Multidimensional Fatigue Inventory, PHQ9: 9-question Patient Health Questionnaire for depression, MoCA: Montréal Cognitive Assessment.
